# A Likelihood-Based SLIC Superpixel Algorithm for SAR Images Using Generalized Gamma Distribution

**DOI:** 10.3390/s16071107

**Published:** 2016-07-18

**Authors:** Huanxin Zou, Xianxiang Qin, Shilin Zhou, Kefeng Ji

**Affiliations:** 1 College of Electronic Science and Engineering, National University of Defense Technology, Changsha 410073, China; slzhou@nudt.edu.cn (S.Z.); jikefeng@nudt.edu.cn (K.J.); 2 School of Information and Navigation, Air Force Engineering University, Xi’an 710077, China; qxxzhijia@126.com

**Keywords:** superpixel, simple linear iterative clustering, likelihood, synthetic aperture radar, generalized gamma distribution, edge evolving

## Abstract

The simple linear iterative clustering (SLIC) method is a recently proposed popular superpixel algorithm. However, this method may generate bad superpixels for synthetic aperture radar (SAR) images due to effects of speckle and the large dynamic range of pixel intensity. In this paper, an improved SLIC algorithm for SAR images is proposed. This algorithm exploits the likelihood information of SAR image pixel clusters. Specifically, a local clustering scheme combining intensity similarity with spatial proximity is proposed. Additionally, for post-processing, a local edge-evolving scheme that combines spatial context and likelihood information is introduced as an alternative to the connected components algorithm. To estimate the likelihood information of SAR image clusters, we incorporated a generalized gamma distribution (GГD). Finally, the superiority of the proposed algorithm was validated using both simulated and real-world SAR images.

## 1. Introduction

Recently, object-based algorithms such as the classification of remote sensing images have become very popular, especially high-resolution (HR) ones [1,2,3,4,5]. Compared to the traditional pixel-based methods, object-based algorithms process images at the regional level instead of at the pixel level, increasing the availability of information. However, additional region generation methods are generally required beforehand, e.g., superpixel algorithms [6,7,8,9].

Superpixel algorithms are methods that group pixels into meaningful atomic regions of similar size [10,11]. Many superpixel algorithms have been developed, including normalized cuts [12], agglomerative clustering [13], quick shift [14] and Turbopixel algorithms [15]. Recently, a superpixel algorithm called simple linear iterative clustering (SLIC) [10,11] has been proposed, which, compared to the state-of-the-art superpixel methods, is superior for both boundary adherence and efficiency. The SLIC has two steps. Firstly, it generates superpixels by grouping pixels with a local k-means clustering (KMC) method, where the distance is measured as the Euclidean distance integrated with the data and spatial distances. Secondly, a connected components algorithm (CCA) is used to remove the generated small isolated regions by merging them into the nearest large superpixels.

The SLIC method has shown good performance for numerous optical images [10,11], but may provide bad superpixels for synthetic aperture radar (SAR) images. The main reasons are as follows. First, SAR images are often corrupted by widespread inherent speckles. Using the standard SLIC method, the generated superpixels often contain various isolated small image regions and the boundaries of these superpixels may deviate significantly from the actual ones. A further complication of these images is that due to the microwave imaging mechanism, SAR images with different scenes are more likely to cover a larger dynamic range of intensity than optical images, especially when the scenes include both natural and man-made terrains. For these types of images, the use of standard SLIC with a fixed regularization parameter balancing the data and spatial distances would generate bad superpixels.

To address the challenges of applying the SLIC method to SAR images, an improved SLIC is proposed in this paper. The use of generalized gamma distribution (GГD) [16] to model SAR images allows utilization of the likelihood information of SAR image pixel clusters. Additionally, an edge evolving scheme (EES) combining the spatial context with likelihood information is applied in the post-processing procedure to remove small isolated regions and improve the boundary adherence of superpixels.

## 2. Standard SLIC Algorithm

The standard SLIC algorithm shows fine performance of generating superpixels for optical images and is applied as follows. Let $N_{p}$ be the number of pixels in a given image and k the number of superpixels to generate. Next, the main steps of the SLIC algorithm are as follows [10,11]:

(1) Initialize cluster centers. Set k initial cluster centers on a regular grid spaced $S=\sqrt{N_{p}/k}$ pixels apart, and then move these cluster centers to the positions with the lowest gradients in a 3 × 3 neighborhood;
(2) Assign pixels. Designate each pixel to a closest cluster center in a local search space by local KMC;
(3) Update cluster centers. Set each cluster center as the mean of all pixels in the corresponding cluster;
(4) Repeat steps (2)–(3) until the clusters do not change or another given criterion is met;
(5) Post-processing. The CCA is used to reassign isolated regions to nearby superpixels if the size of the isolated regions is smaller than a minimum size $S_{\min}$.

**Figure 1:**
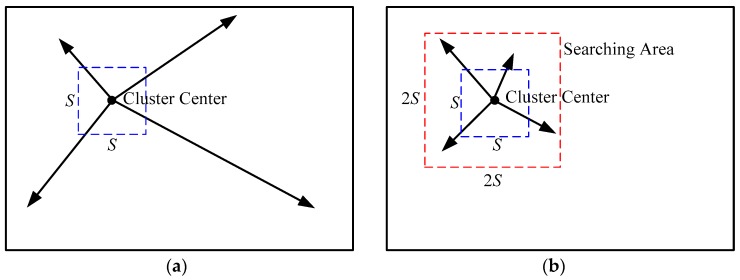
Illustrations of search areas of cluster center in (**a**) conventional k-means clustering (KMC) and (**b**) local KMC used in the simple linear iterative clustering (SLIC) algorithm.

A local KMC is applied in step (2) of the SLIC method, where each pixel is associated with the closest cluster center whose search area covers its location. Figure 1 illustrates the search area of a cluster center using conventional KMC or the local KMC used in the SLIC algorithm [10,11]. In conventional KMC, the search area of each cluster center is the whole image, and then the distances are calculated from each cluster center to every pixel in the image. In local KMC, however, the search space of a cluster center is limited to a local 2*S* × 2*S* square region. Therefore, the SLIC only computes distances from each cluster center to pixels within its searching area. 

In local KMC, Euclidean distance is used in the clustering. Let $z_{i}$ be the data (although standard SLIC was originally designed for color optical images, for SAR images, only the intensity is considered) of the *i*-th cluster center with its spatial position as $\left(x_{i},y_{i}\right)$. Let $z_{j}$ be the intensity of a pixel within the search area of the center. Then, the integrated distance between this pixel and the center is:

(1) $$D_{I}=\sqrt{{\left(d_{f}/m\right)}^{2}+{\left(d_{s}/S\right)}^{2}}$$

where $d_{f}=\left|z_{i}-z_{j}\right|$ and $d_{s}=\sqrt{{\left(x_{i}-x_{j}\right)}^{2}+{\left(y_{i}-y_{j}\right)}^{2}}$ are the intensity and spatial distances between the pixel and the center, respectively, and m is a regularization parameter that weights the relative contribution of $d_{f}$ and $d_{s}$ to the integrated distance $D_{I}$. A larger m indicates that $d_{s}$ is more significant than $d_{f}$. An equivalent integrated distance $D_{I}$ directly describing the contribution of the two distances can be given by:

(2) $$D_{I}=\sqrt{w{\left(d_{f}/N_{f}\right)}^{2}+\left(1-w\right){\left(d_{s}/S\right)}^{2}}$$

where $N_{f}$ is the mean intensity of the whole image, w∈[0,1] is a regularization parameter. In this context, w and (1−w) are the ratios of the normalized intensity and spatial distances in $D_{I}$, respectively.

## 3. Proposed Likelihood-Based Superpixel Algorithm

### 3.1. Local Likelihood-Based Clustering

In the standard SLIC method, the spatial and intensity distances influence the regularity and boundary adherence of superpixels, respectively. For intensity distance, only the mean intensity is used for each image cluster as a representative statistic for various optical images. However, this may be insufficient to describe SAR image clusters due to the inherent speckle noise. Therefore, to characterize SAR image clusters, the likelihood information is generally more useful than the mean. 

**Figure 2:**
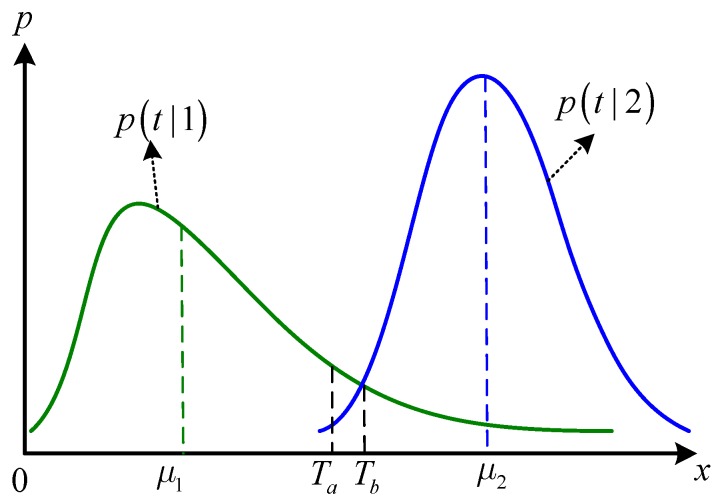
Illustration of decision thresholds in k-means and maximum likelihood (ML) methods.

To visually compare the mean and the likelihood value used in the algorithms, Figure 2 illustrates decision thresholds used in the general k-means method and maximum likelihood (ML) classifier for a binary classification problem. In each algorithm, a decision threshold is first determined, and then the pixels with intensities smaller than the threshold are assigned to the first class, otherwise, the other one. In Figure 2, p(t|1) and p(t|2) are the conditional probability density functions (PDFs) of two categories with their means as $\mu_{1}$ and $\mu_{2}$, respectively. Then, the decision threshold $T_{a}$ of the *k*-means method is $T_{a}=\left(\mu_{1}+\mu_{2}\right)/2$, and the threshold $T_{b}$ of ML classifier corresponds to the crosspoint of the two PDFs. 

Assume the prior probability is equal for each of these two classes, then for a given decision threshold *T*, the classification error can be computed by [17]:

(3) $$P_{e}=\frac{1}{2}\left\{\int_{T}^{\infty }p\left(t|1\right)dt+\int_{-\infty }^{T}p\left(t|2\right)dt\right\}$$

Then, the threshold $T=T_{b}$, which corresponds to the crosspoint of the two PDFs, would lead to a smallest decision error [17]. If the clusters have symmetric PDFs, such as a Gaussian distribution, then the threshold of k-means method would be equal to that of the ML algorithm, namely $T_{a}=T_{b}$, thus yielding a smaller decision error. In practice, however, the PDFs of various SAR image clusters are generally asymmetric, especially for heterogeneous areas such as urban areas. For analysis of these images, $T_{a}$ generally deviates more from $T_{b}$, resulting in a larger decision error. To account for this, we introduced a likelihood value to assess the distance or similarity between each pixel and cluster of SAR images. Generally, a similarity measure is a real-valued function that quantifies the similarity between two objects. 

In our clustering method, we maintained the spatial distance $d_{s}$ used in the standard SLIC method but modified the intensity distance based on the above analysis. Since the spatial distance is independent of the data dynamic of SAR images, we first normalized the dynamic range of the given SAR image to form a proper clustering scheme. To segment a given SAR image $I_{0}$, it is first normalized by dividing by its mean $\mu_{I}$, so $I=I_{0}/\mu_{I}$. Additionally, the likelihood value rather than the Euclidean distance is adopted to represent the intensity similarity between a pixel and a cluster. 

Let p(t|i) be the conditional PDF of the *i*-th cluster $R_{i}$, then, with respect to a given pixel with the intensity as $z_{j}$, we can get the likelihood value of cluster $R_{i}$ as $L=p\left(z_{j}|i\right)$. To combine this likelihood value with the spatial distance, an intensity similarity $S_{f}$ and a spatial proximity $S_{d}$ between the pixel with the intensity $z_{j}$ and the cluster $R_{i}$ are defined, respectively, as follows:

(4) $$S_{f}=1-\exp\left\{-L\right\}=1-\exp\left\{-p\left(z_{j}|i\right)\right\}$$

(5) $$S_{d}=1-\exp\left\{-1/\left(d_{s}/S\right)\right\}$$

In this context, an integrated similarity measure between the pixel and the cluster $R_{i}$ can accordingly be defined and is shown as follows:

(6) $$S_{I}=wS_{f}+\left(1-w\right)S_{d}$$

The domain of $S_{I}$ is $S_{I}\in \left(0,1\right]$. A larger $S_{I}$ value indicates a higher similarity between the pixel and the cluster. In our local clustering scheme, each pixel is assigned to a nearest cluster with the maximum similarity $S_{I}$, instead of the minimum distance $D_{I}$ in the standard SLIC method. For simplicity, this clustering is named likelihood-based clustering (LC).

### 3.2. Local Likelihood-Based Edge Evolving

After local clustering, some small isolated regions may be generated. In the standard SLIC method, each small region must be merged into a large nearby superpixel by the CCA. Though this manipulation is easy to perform, it may also result in worse edges or boundaries of superpixels. Since some small regions may be very different from adjacent regions, forced merging by the CCA would lead to bad results. As an alternative, we introduced a local likelihood-based edge evolving scheme (EES) based on our similar related work [18]. This strategy deals with the generated small regions differently than the CCA method, and the removal of these regions is not mandatory. The basic idea of EES is to adjust the edges of the previous superpixels iteratively by combining spatial context and likelihood information to improve the boundary adherence and remove the small regions that are mainly caused by the influence of speckle. The EES is described as follows.

Let G be the coordinate set of image pixels, $l=\left\{l_{g}\in \left\{1,2,...,M\right\}|g\in G\right\}$ is the segmentation with M superpixels $\Omega_{1},...,\Omega_{M}$, and $z_{b}$ is the intensity of an arbitrary edge pixel of the superpixels. The joint probability distribution $P\left(l_{b},z_{b}\right)$ can be written in two different ways: $P\left(l_{b},z_{b}\right)=P\left(l_{b}|z_{b}\right)p\left(z_{b}\right)=p\left(z_{b}|l_{b}\right)P\left(l_{b}\right)$, leading to the Bayes’ formula as [17]:

(7) $$P\left(l_{b}|z_{b}\right)=\frac{p\left(z_{b}|l_{b}\right)P\left(l_{b}\right)}{p\left(z_{b}\right)}$$

where $P\left(l_{b}|z_{b}\right)$ and $P\left(l_{b}\right)$ are the a posterior and priori probabilities of the edge pixel’s segmentation label $l_{b}\in \left\{1,2,...,M\right\}$, respectively, $p\left(z_{b}|l_{b}\right)$ is the likelihood value or conditional PDF of the edge pixel with respect to $\Omega_{l_{b}}$, and $p\left(z_{b}\right)$ is the PDF of the edge pixel. 

In our method, to adjust the previous superpixels’ edge pixels, the maximum a posterior (MAP) criterion is applied. Since $p\left(z_{b}\right)$ is independent of the segmentation label $l_{b}$, the edge pixel’s segmentation label is reassigned by:

(8) $${\hat{l}}_{b}=\underset{l_{b}\in \left\{1,2,...M\right\}}{\arg\max}\left\{P\left(l_{b}|z_{b}\right)\right\}=\underset{l_{b}\in \left\{1,2,...M\right\}}{\arg\max}\left\{p\left(z_{b}|l_{b}\right)P\left(l_{b}\right)\right\}$$

Generally, it is assumed that the image’s segmentation label field l satisfies the Markovianity. In other words, it is supposed that l is an Markov random field (MRF) on G with respect to a neighborhood system $\eta_{g}$ satisfying two conditions, e.g., p(l)>0 and $p\left(l_{g}|l_{Gg}\right)=p\left(l_{g}|l_{\eta_{g}}\right)$, where Gg is the set difference of G except g, $l_{Gg}$ and $l_{\eta_{g}}$ are the label sets on Gg and $\eta_{g}$, respectively [18,19]. Then, according to the Hammersley-Clifford theorem that an MRF is equivalent to a Gibbs random field (GRF) [19], the local probability $P\left(l_{b}|l_{\eta_{b}}\right)$ can be computed by the Gibbs probability as:

(9) $$P\left(l_{b}|l_{\eta_{b}}\right)=Z^{-1}\exp\left\{-\sum_{c\in \eta_{b}}V_{c}\left(l_{b}\right)\right\}$$

where $V_{c}\left(l_{b}\right)$ is the potential function, c is a clique in the neighborhood $\eta_{b}\in G$, and Z is the normalization coefficient. In order to compute $V_{c}\left(l_{b}\right)$, an isotropic second order neighborhood system and the related set of pair-site cliques are utilized (for more details, the reader is referred to [20]). Then, by replacing $P\left(l_{b}\right)$ with $P\left(l_{b}|l_{\eta_{b}}\right)$ in Equation (8), the criterion to reassign edge pixel becomes:

(10) $${\hat{l}}_{b}=\underset{l_{b}\in l_{\eta_{b}}}{\arg\max}\left\{p\left(z_{b}|l_{b}\right)P\left(l_{b}|l_{\eta_{b}}\right)\right\}$$

In the EES procedure described in our earlier work [18], an ML edge-evolving loop was performed before the MAP loop because of the bad estimation of edges’ labels. However, in our algorithm, the previous local likelihood clustering procedure provided good estimation of such labels, so the EES can be briefly performed only with the MAP loop. This is performed according to the following steps:

(1) Estimate the likelihood of pixel intensities within each superpixel and count the total number of edge pixels of all superpixels, denoted by $N_{b}$;
(2) Reassign each edge pixel by Equation (10), and count the total number of edge pixels whose labels have been changed, denoted by $N_{c}$;
(3) Compute the change rate of the edge pixels defined by $R_{c}=N_{c}/N_{b}$, indicating the ratio of the number of edge pixels that were modified to the number of total edge pixels;
(4) Repeat steps (1)–(3) until $R_{c}$ reaches a prespecified small value, i.e., $R_{T}$.

### 3.3. Statistical Modeling of SAR Images by GГD

As seen previously, in our proposed algorithm, it is important to estimate the likelihood or conditional PDF of each cluster of the SAR images, for which the statistical modeling of SAR images is critical. The parametrical method is widely used to estimate the conditional PDF of SAR images, in which a parametrical model is specified to the SAR image beforehand, and the corresponding parameters of the model are then estimated with the given image data samples.

The generalized Gamma distribution (GГD) is a famous parametrical model first proposed by Stacy [21] and subsequently applied in many fields such as speech and image processing. This model exhibits high flexibility and superior performance to many classical distributions for modeling SAR images [16,21]. Additionally, it can be reduced to many SAR image models, including the Exponential, Weibull, Gamma, inverse Gamma, and log-normal distributions.

In our method, GГD is employed for the statistical modeling of SAR image clusters. The GГD is a distribution defined by [16]:

(11) $$p\left(z\right)=\frac{\left|\nu \right|\kappa^{\kappa }}{\sigma \Gamma \left(\kappa \right)}{\left(\frac{z}{\sigma }\right)}^{\kappa \nu -1}\exp\left\{-\kappa {\left(\frac{z}{\sigma }\right)}^{\nu }\right\},\sigma ,\left|\nu \right|,\kappa ,z>0$$

where σ, ν and κ are the scale, power, and shape parameters, respectively. 

For the parameter estimation of GГD, the method of log-cumulants (MoLC) [22,23] can be employed. This approach estimates parameters by solving an equation system of log-cumulants. The first three log-cumulants of GГD can be computed as [16]:

(12) $$\left\{ \begin{array}{l} c_{1}=\ln\sigma +\left(\Psi \left(\kappa \right)-\ln\kappa \right)/\nu \\ c_{i}=\Psi \left(i-1,\kappa \right)/\nu^{i},i=2,3 \end{array}\right.$$

where Ψ(x)=dlnΓ(x)/dx is the digamma function and $\Psi \left(i,x\right)=d^{i}\Psi \left(x\right)/dx^{i}$ is the *i*-th order polygamma function. In practice, the log-cumulants would be replaced by empirical ones. For more details, the reader is referred to [16,22,23].

In the two main steps of our algorithm, namely the clustering and edge evolving steps, the statistical modeling procedures are required for each SAR image region. Let $Z=\left\{Z_{1},Z_{2},...,Z_{N}\right\}$ be the intensity data of an arbitrary SAR image region consisting of *N* pixels. It is assumed that Z follows the GГD as shown in Equation (11). Then, the first three empirical log-cumulants of Z can be calculated by:

(13) $$\left\{ \begin{array}{l} {\hat{c}}_{1}=\frac{1}{N}\sum_{n=1}^{N}\ln Z_{n}\\ {\hat{c}}_{t}=\frac{1}{N}\sum_{n=1}^{N}{\left(\ln Z_{n}-{\hat{c}}_{1}\right)}^{t},t=2,3 \end{array}\right.$$

Thus, by replacing these three empirical log-cumulants with the theoretical ones in Equation (12) and solving the equations, the parameters σ, ν, and κ of GГD for the data Z are estimated. In other words, the PDF of Z is estimated. 

## 4. Experiments and Discussion

### 4.1. Evaluation on Simulated SAR Image

**Figure 3:**
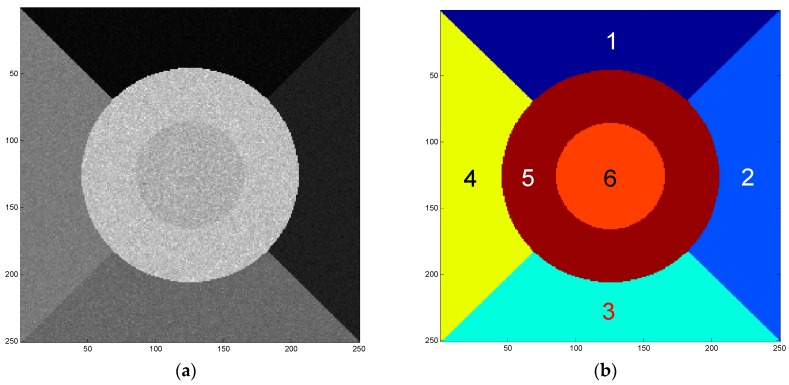
(**a**) Simulated synthetic aperture radar (SAR) image with six regions following the generalized gamma distributions (GГDs) and (**b**) the corresponding ground truth.

In our experiments, the SAR images are assumed to follow GГD. To evaluate our proposed algorithm quantitatively, a simulated SAR image with six regions following the GГDs is first generated by a non-linear transformation method [24] as shown in Figure 3a. It consists of 250 × 250 pixels and its ground truth is given in Figure 3b. The parameters {σ,ν,κ} of GГD corresponding to each of the six regions numbered by 1–6 are {5,4,8}, {8,4,8}, {40,−2,8}, {60,−2,8}, {200,−1,8} and {300,−1,8}, respectively. These six regions can be divided into three pairs from the viewpoint of intensity, namely regions 1 and 2 are the low-value region pair, regions 3 and 4 the median-value pair, and regions 5 and 6 are the high-value ones. 

Next, three methods were evaluated for the simulated SAR image, including standard SLIC, the proposed method, and a compound method that combines LC with the CCA. For these methods, the superpixel size S was set as 20 and a sequence of regularization parameters were set as w={0.1,0.2,...,1}. Additionally, $S_{\min}=12$ in the CCA and $R_{T}=0.01$ in the EES, indicating that the labels of more than 99 percent of edge pixels remained unchanged. To evaluate the results quantitatively, the boundary recall (BR) and the under-segmentation error (USE) were also applied, which are defined as follows.

1. Boundary recall: The BR computes what fraction of ground truth edges overlap exactly with the boundary pixels of the obtained superpixels, and is computed by:
  (14) $$BR=N_{\text{GT}\cap \text{SP}}/N_{\text{GT}}$$
  where $N_{\mathrm{GT}\cap \mathrm{SP}}$ is the number of boundary pixels shared by the ground truth and the obtained superpixels, and $N_{\mathrm{GT}}$ denotes the number of boundary pixels of the ground truth. In our work, the internal boundaries of ground truth and superpixels are used.
2. Under-segmentation error: Given ground truth segments $g_{1},g_{2},...,g_{M}$ and a superpixel output $s_{1},s_{2},...,s_{L}$, the under-segmentation error is defined by [10,11]:
  (15) $$USE=\frac{1}{N}\left[\sum_{i=1}^{M}\left(\sum_{\left[s_{j}|s_{j}\cap g_{i}>B\right]}\left|s_{j}\right|\right)-N\right]$$
  where, |⋅| gives the size of the segment in pixels, N is the size of the image in pixels, the expression $s_{j}\cap g_{i}$ is the intersection or overlapping error of a superpixel $s_{j}$ with respect to a ground truth segment $g_{i}$, and B denotes a minimum number of pixels in $s_{j}$ overlapping $g_{i}$.

**Figure 4:**
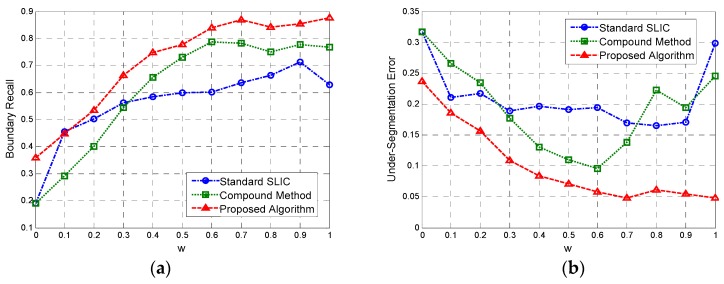
(**a**) boundary recall curves and (**b**) under-segmentation error plots obtained by three methods tested on the simulated SAR image.

**Figure 5:**
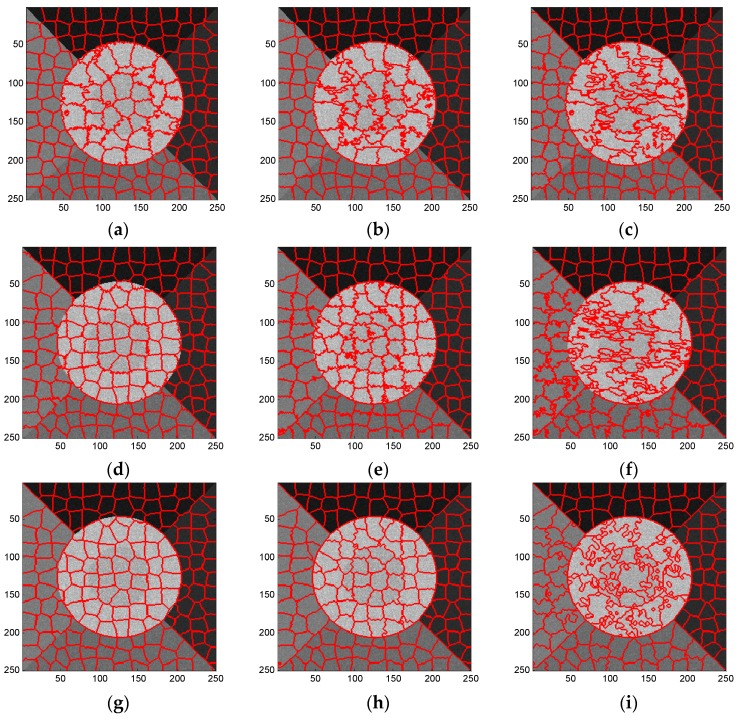
Superpixels of simulated SAR image provided by (**a**–**c**) standard SLIC algorithm; (**d**–**f**) compound method; and (**g**–**i**) proposed algorithm with regularization parameter w set to 0.3, 0.6 and 0.9, from left to right.

Figure 4a,b illustrate the BR and USE provided by these three methods, respectively. Some results of representative superpixels are shown in Figure 5. The results from top to bottom refer to the standard SLIC, Figure 5a–c; compound method, Figure 5d–f; and proposed algorithm, Figure 5g–i; and those from left to right correspond to the regularization parameter w set to 0.3, 0.6 and 0.9, respectively.

It can be seen from Figure 4 that the BR curves obtained by the standard SLIC and the compound methods cross each other, and the latter has a larger maximum value than the former. Similarly, the lowest USE obtained by the compound method is much smaller than that of the standard SLIC. These results validated the superiority of the proposed LC scheme compared to the KMC method used in the standard SLIC. Moreover, the BR (USE) plot of the results generated by the proposed algorithm is consistently higher (lower) than the results obtained using the compound algorithm. These findings suggest that the EES model is more accurate than the CCA for these two quantitative criteria. 

**Figure 6:**
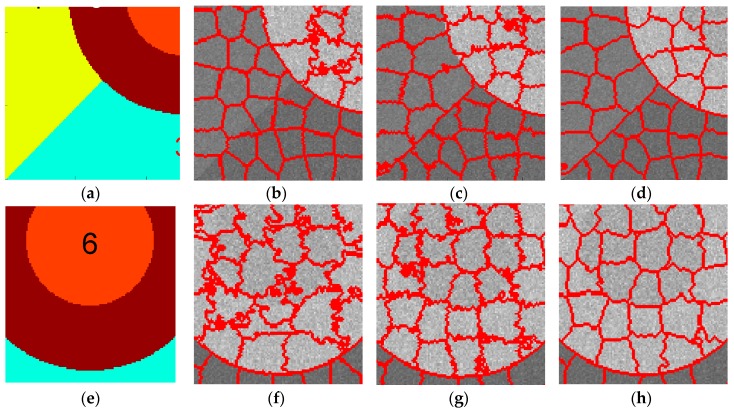
Ground truth of simulated image patches 1 and 2 (**a**,**e**) and corresponding superpixels provided by standard SLIC (**b**,**f**), compound method (**c**,**g**) and proposed algorithm (**d**,**h**).

Overall, as shown in Figure 5, the proposed algorithm yields better superpixel results than the other two methods. For the small regularization parameter, the standard SLIC generates good boundaries only between the high-value regions and other regions. In contrast, the compound method recalls better boundaries between low and median-value regions, while the proposed algorithm provides the best edges, even for those between regions with little contrast, e.g., regions 1 and 2. Additionally, with the increase of regularization parameter, the three algorithms yield superpixels with more irregular shapes. Therefore, in practice, a medium regularization parameter such as 0.5 or 0.6 can be adopted in our method to obtain a good trade-off between boundary adherence and the regularity of superpixels. With a medium regularization parameter of w, 0.6, Figure 6 illustrates the edge details yielded by the three algorithms focusing on two sub-regions of the simulated image. It can be clearly observed from Figure 6 that the compound method catches the actual boundaries between regions 3 and 4 (Figure 6b–d) and between regions 5 and 6 (Figure 6f–h) better than the original SLIC algorithm, and the proposed algorithm recalls these actual boundaries more accurately. These results further demonstrate the superior performance of the proposed algorithm. 

Next, the efficiencies of these three methods were compared. Without loss of generality, Table 1 reports the time cost for different parts of the three methods for w=0.6 performed on the simulated SAR image. The experiments were performed by Matlab codes running on the same computer with a 2.0 GHz CPU and 3.0-GB memory. 

The LC involves more computational time than the KMC, about 2.4 times. Compared to the time cost in the two clustering steps, due to the simplicity of CCA, the time consumed in the CCA is minimal. By comparison, the EES provides better superpixels, but this is more time-consuming, about one more order of magnitude than the cost in the clustering procedure. Therefore, in practice, EES is recommended for post-processing when the segmentation quality is more important than efficiency, otherwise, CCA is suggested to be used. 

### 4.2. Evaluation on Real-World SAR Images

**Figure 7:**
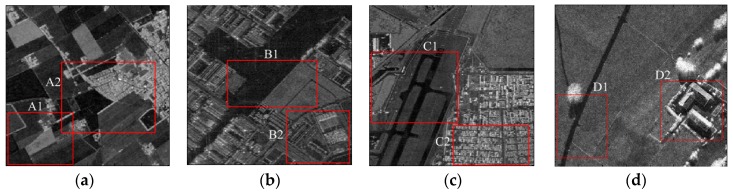
(**a**) ElectroMagnetic Institute Synthetic Aperture Radar (EMISAR) image; (**b**) Airborne Synthetic Aperture Radar (AIRSAR) image; (**c**) Uninhabited Aerial Vehicle Synthetic Aperture Radar (UAVSAR) image; and (**d**) Flugzeug Synthetic Aperture Radar (F-SAR) image.

To further evaluate the performance of our proposed method, we next analyzed four real-world SAR intensity images as shown in Figure 7. Figure 7a–d show the images acquired by the ElectroMagnetic Institute Synthetic Aperture Radar (EMISAR), Airborne Synthetic Aperture Radar (AIRSAR), Uninhabited Aerial Vehicle Synthetic Aperture Radar (UAVSAR) and Flugzeug Synthetic Aperture Radar (F-SAR) systems, respectively. The detailed parameters (including the polarization, band, size, resolution, acquisition location and acquisition year) of the four SAR images are listed in Table 2. These four SAR images depict various terrains with a large dynamic range of intensity, including farmlands, water, grassland, trees, and building areas.

**Figure 8:**
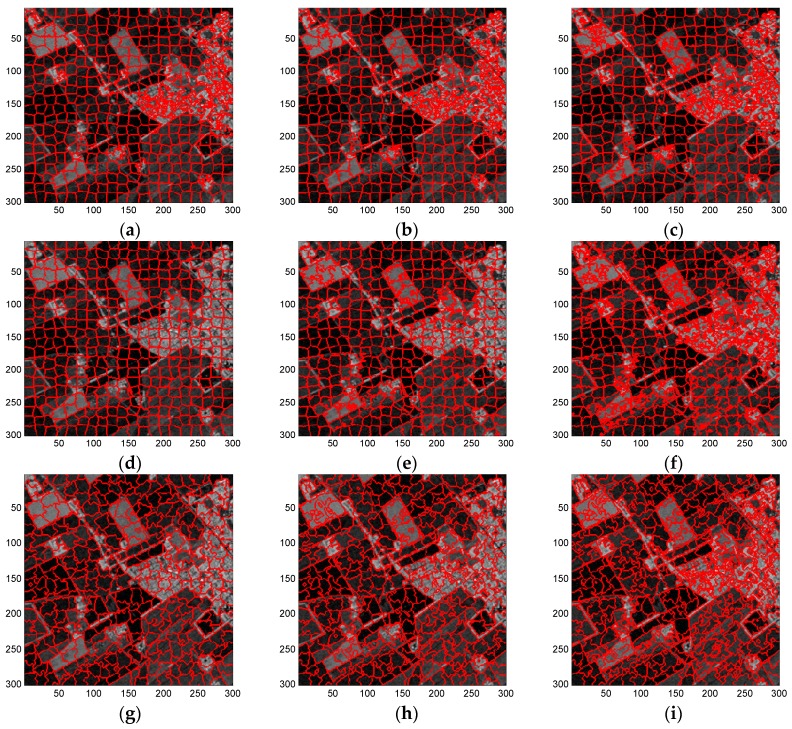
Superpixels of EMISAR image yielded by (**a**–**c**) standard SLIC algorithm; (**d**–**f**) compound method; and (**g**–**i**) proposed algorithm with regularization parameter w as 0.3, 0.6, and 0.9 from left to right.

The standard SLIC, the compound method, and proposed algorithm were performed separately on these images. In the superpixel generation algorithm, the superpixel size is generally set according to the complexity of the underlying SAR image empirically or set to meet a certain requirement. In our experiments, the superpixel size was set as 12 for the UAVSAR image, and as 15 for the other three SAR images. $S_{\min}$ was set as 7 empirically in the CCA and $R_{T}=0.01$ in the EES, indicating that the edge-evolving step stops when less than 1 percent of the labels of edge pixels were changed in the previous loop. Figure 8 illustrates the generated superpixels of the EMISAR image after application of these three methods with three representative regularization parameters as w=[0.3,0.6,0.9]. Due to the absence of the ground truth for real-world SAR images, the performance of these three algorithms were evaluated and compared by visual observation and inspection, with a particular focus on the definition of the boundary between terrains. 

**Figure 9:**
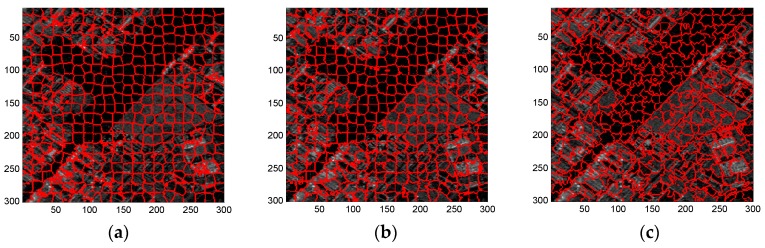
Superpixels of AIRSAR image provided by (**a**) standard SLIC; (**b**) compound method; and (**c**) proposed algorithm with regularization parameter w=0.6.

**Figure 10:**
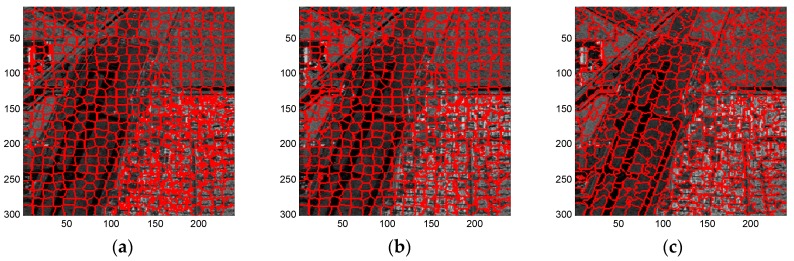
Superpixels of UAVSAR image provided by (**a**) standard SLIC; (**b**) compound method; and (**c**) proposed algorithm with regularization parameter w=0.6.

**Figure 11:**
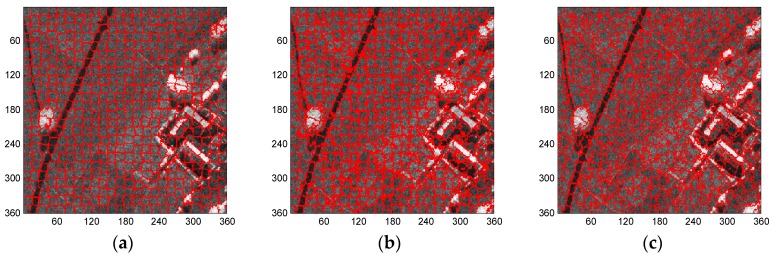
Superpixels of F-SAR image provided by (**a**) standard SLIC; (**b**) compound method; and (**c**) proposed algorithm with regularization parameter w=0.6.

As can be seen from Figure 8, similar to the results using the simulated SAR image, each of these three methods provides superpixels that adhere well to the real terrain boundaries but with less regular shapes when the regularization parameter w increases gradually. In general, a moderate regularization parameter such as w=0.6 can be a good compromise between boundary adherence and the regularity of superpixels. Therefore, for simplicity, Figure 9, Figure 10 and Figure 11 provides the superpixels of the AIRSAR, UAVSAR, and F-SAR images, respectively, provided by these three methods with a fixed regularization parameter w=0.6.

**Figure 12:**
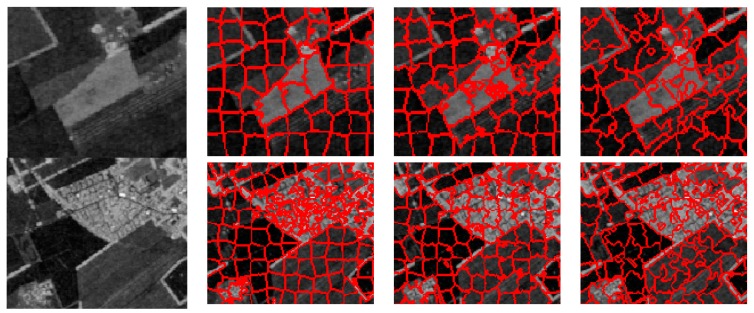
EMISAR image patches A1 and A2 (first column from top to bottom) and corresponding superpixels provided by standard SLIC (second column), compound method (third column), and proposed algorithm (fourth column).

**Figure 13:**
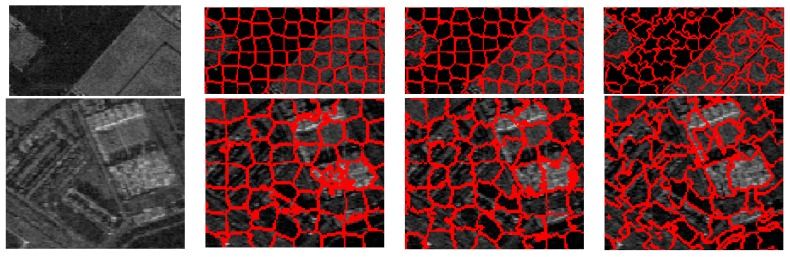
AIRSAR image patches B1 and B2 (first column from top to bottom) and corresponding superpixels provided by standard SLIC (second column), compound method (third column), and proposed algorithm (fourth column).

**Figure 14:**
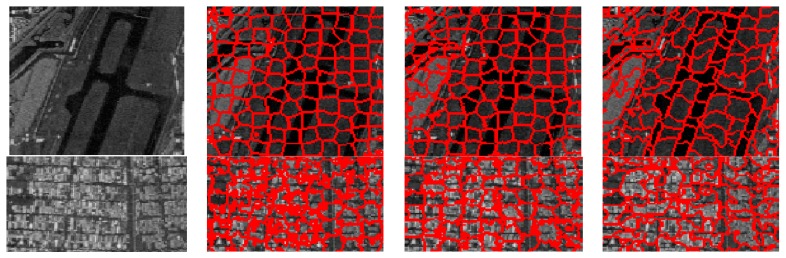
UAVSAR image patches C1 and C2 (first column from top to bottom) and corresponding superpixels provided by standard SLIC (second column), compound method (third column), and proposed algorithm (fourth column).

**Figure 15:**
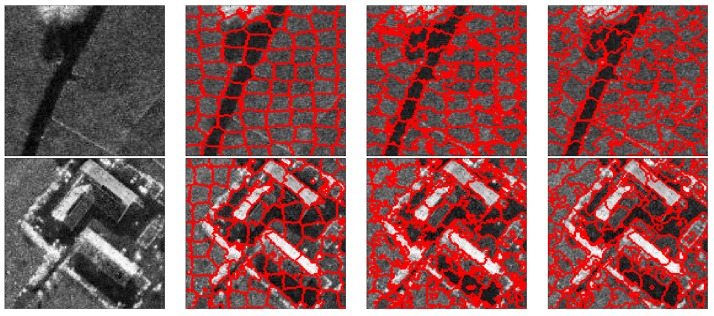
F-SAR image patches D1 and D2 (first column from top to bottom) and corresponding superpixels provided by standard SLIC (second column), compound method (third column), and proposed algorithm (fourth column).

To further evaluate the performance of these three methods for the generation of superpixels in the SAR images, some typical superpixel results of two portions of each SAR image are shown for detailed comparison. Figure 12, Figure 13, Figure 14 and Figure 15 present the results of typical image patches of the EMISAR, AIRSAR, UAVSAR, and F-SAR images, respectively, and indicated by rectangles A1 and A2 in Figure 7a, B1 and B2 in Figure 7b, C1 and C2 in Figure 7c, and D1 and D2 in Figure 7d. The three methods were performed with a fixed regularization parameter w=0.6.

By comparing the superpixels of the four images generated by different methods, it is easily observed that the compound method provides generally better results than the standard SLIC algorithm, and our proposed algorithm yields overall best results. The standard SLIC algorithm produces good results between terrains with large contrast, but poor results for many terrains with small contrast, e.g., the farmlands in the regions A1 and A2 of the EMISAR image, regions B1 and B2 of the AIRSAR image, patches C1 and C2 of the UAVSAR image, and areas D1 and D2 of the F-SAR image, where many real edges were not recalled correctly. By comparison, the compound method captures the real edges better than the standard SLIC. This is mainly because the LC utilizes more cluster information than the KMC and is more robust for the large dynamic range of SAR images. 

Additionally, it can be visually observed that the proposed algorithm provides superpixels with boundaries that are closest to the real ones, even for the terrains whose width is smaller than the given size of the superpixels. For examples, the superpixels yielded by the proposed method recall the real edges of some building (as in regions B2, C2 and D2) and some road areas (as in regions C1 and D1) unlike the other two approaches. The EES is superior to the CCA for capturing the real terrain edges because in the CCA approach, small isolated regions are eliminated by simply merging them into the large nearby superpixels but most edge pixels of superpixels are maintained, even though they may substantially deviate from the real ones. In contrast, using EES, all the edge pixels of superpixels are fully considered for adjustment according to the spatial context information, resulting in better adherence of the generated superpixels to the real terrains. 

In addition, to compare the efficiencies of these three algorithms, without loss of generality, the time cost by these methods with w=0.6 on the EMISAR image is reported in Table 3. 

As shown in Table 3, the disadvantage of the EES is that it is much more time consuming than the CCA, about one order of magnitude higher than the previous LC step. Therefore, it would be valuable to improve the efficiency of EES, which was not considered here and will be the focus of future studies.

## 5. Conclusions

In this paper, a likelihood-based SLIC superpixel algorithm for SAR images was proposed. Compared to the standard SLIC, our proposed algorithm performs a local likelihood-based clustering (LC) instead of k-means clustering (KMC). In LC, a similarity criterion was designed to combine intensity distance and the spatial proximity. Moreover, an edge-evolving scheme (EES) was introduced for post-processing to achieve fine boundary adherence instead of the connected components algorithm (CCA) used in the standard method. In addition, GГD was employed to model the generated clusters of SAR images. Extensive experiments performed on both simulated and real-world SAR images demonstrate that the proposed LC is more robust to the large dynamic range of SAR images, and thus could generate superpixels that recall actual boundaries with greater accuracy and with a lower under-segmentation error than KMC. Additionally, we found that the EES model allowed improved boundary adherence compared to the CCA. In other words, by using the EES approach, the obtained superpixel edges correspond better to the edges between actual terrains than the superpixel edges obtained using the CCA method. However, due to the additional operations required, the EES is more time-consuming than the CCA. Therefore, it would be beneficial to improve the efficiency of the EES method in future work. 

## Figures and Tables

**Table 1:** Time (in seconds) of the three superpixel algorithms for w=0.6 on the simulated synthetic aperture radar (SAR) images.

Algorithm	Clustering		Post-Processing		Total Time
	Scheme	Time	Scheme	Time	
Standard SLIC	KMC	3.288	CCA	0.073	3.361
Compound method	LC	7.957	CCA	0.074	8.031
Proposed algorithm	LC	7.957	EES	56.754	64.711

**Table 2:** Parameters of four SAR images used in experiments, where HV, HH and VV denote the polarization models for horizontal transmit and vertical receive, horizontal transmit and horizontal receive, and vertical transmit and vertical receive, respectively.

Figure Number	System	Polarization	Band	Size (Pixels)	Resolution	Acquisition Location	Acquisition Year
Figure 7a	EMISAR	HV	L	300 × 300	1.5 m × 0.75 m	Foulum, Denmark	1998
Figure 7b	AIRSAR	VV	C	300 × 300	13.5 m × 5.5 m	Tokyo, Japan	2000
Figure 7c	UAVSAR	HH	C	300 × 240	1.67 m × 0.6 m	Gulf Coast, America	2011
Figure 7d	F-SAR	HH	C	360 × 360	0.6 m × 0.6 m	Kaufbeuren, Germany	2009

**Table 3:** Time (in second) of three superpixel algorithms with w=0.6 on ElectroMagnetic Institute Synthetic Aperture Radar (EMISAR) images.

Algorithm	Clustering		Post-Processing		Total Time
	Scheme	Time	Scheme	Time	
Standard SLIC	KMC	12.668	CCA	0.112	12.780
Compound method	LC	23.253	CCA	0.128	23.381
Proposed algorithm	LC	23.253	EES	292.439	315.692
